# From the Child’s Word to Clinical Intervention: Novel, New, and Innovative Approaches to Symptoms in Pediatric Palliative Care

**DOI:** 10.3390/children5040045

**Published:** 2018-03-28

**Authors:** Katharine E. Brock, Joanne Wolfe, Christina Ullrich

**Affiliations:** 1 Aflac Cancer and Blood Disorders Center of Children’s Healthcare of Atlanta, Atlanta, GA 30322, USA; Katharine.brock@choa.org; 2 Pediatric Palliative Care, Children’s Healthcare of Atlanta, Atlanta, GA 30322, USA; 3 Department of Pediatrics, Division of Pediatric Hematology/Oncology, Emory University, Atlanta, GA 30322, USA; 4 Department of Psychosocial Oncology and Palliative Care, Dana-Farber Cancer Institute, Boston, MA 02215, USA; Joanne_Wolfe@dfci.harvard.edu; 5 Department of Pediatric Hematology/Oncology, Dana-Farber Cancer Institute, Boston Children’s Hospital Cancer and Blood Disorders Center, Boston, MA 02215, USA

**Keywords:** pediatric palliative care, quality of life, symptom management, hospice, patient-reported outcomes, mobile apps

## Abstract

Despite vast improvements in disease-based treatments, many children live with life-threatening disorders that cause distressing symptoms. These symptoms can be difficult to comprehensively assess and manage. Yet, frequent and accurate symptom reporting and expert treatment is critical to preserving a patient’s physical, psychological, emotional, social, and existential heath. We describe emerging methods of symptom and health-related quality-of-life (HRQOL) assessment through patient-reported outcomes (PROs) tools now used in clinical practice and novel research studies. Computer-based and mobile apps can facilitate assessment of symptoms and HRQOL. These technologies can be used alone or combined with therapeutic strategies to improve symptoms and coping skills. We review technological advancements, including mobile apps and toys, that allow improved symptom reporting and management. Lastly, we explore the value of a pediatric palliative care interdisciplinary team and their role in assessing and managing distressing symptoms and minimizing suffering in both the child and family. These methods and tools highlight the way that novel, new, and innovative approaches to symptom assessment and management are changing the way that pediatrics and pediatric palliative care will be practiced in the future.

## 1. Introduction

Over 400,000 US children live with life-threatening or chronic, complex conditions [1,2]. Over 12,400 children are diagnosed with cancer annually in the United States [3]. Despite vast improvement in overall survival, less attention has been focused on comprehensively assessing and treating symptoms. Even with a bevy of new cancer-directed therapies, children continue to suffer from distressing symptoms such as pain, fatigue, nausea/vomiting, decreased appetite, anxiety, and depression [4,5,6]. Symptoms may arise from the disease itself, or commonly from the treatments prescribed. Many of the symptoms children suffer through are experienced in the home setting, away from the hospital, contributing to the perception of few treatment options [7]. Numerous factors lead to poor symptom control at the patient, parent, healthcare team, and system levels [8]. These include a lack of provider time and skill in addressing symptoms, infrequent use of systematic assessment tools, and provider uncertainty around the accuracy of pediatric patient-reported symptoms [8,9]. Patient forgetfulness, the desire to be a “good patient,” and the belief that one needs to experience symptoms for better chance of cure also impact symptom reporting [10,11]. System challenges include lack of sufficient integrative medicine, chronic pain, psychiatry, psychology, and palliative care resources to meet patients’ needs [12,13,14]. Multiple interventions have been attempted to improve the reporting of symptoms by patients and families, such as improved outpatient screening [15], frequent monitoring [8,16,17], increased use of patient-reported outcomes (PROs) [5,18], technological advances [8,16,17], and embedding palliative care experts within oncology centers [19]. In this article, we describe the current state of symptom assessment and management within pediatric palliative care, with a focus on pediatric oncology. We also describe emerging and novel approaches to symptom assessment for patients with serious and complex illness.

## 2. Technological Advances for Symptom Assessment, Reporting, and Management

Technology use in America is ubiquitous. Ninety-five percent of Americans own a cellphone [20]; 70% own a smartphone, up from 35% in 2011 [20]. Of US adults aged 18–29 years, 100% own a cellphone, with 92% owning a smartphone [20]; among 12–17 year-olds, nearly three-quarters own a smartphone [21]. Along with mobile phones, Americans own a range of other devices; nearly 80% of US adults own a computer, and 51% own tablet devices [20]. Roughly three-quarters have internet access in the home [22]. Of teens and adults ages 18–29 years, nearly 100% use the internet regularly [21,22], and 71–86% are social media users, often accessing sites daily [21,23]. Notably, the use of these devices has extended into the healthcare setting. Technology has changed how patients and clinicians communicate with each other outside of the hospital and how patients or parents connect with other families online. Patients utilize technology to remember appointments, instructions, and enhance their medication adherence [24,25].

As technology plays a larger role in children, patients, and families lives, hospitals, clinicians, and researchers have attempted to utilize technology for symptom assessment and integrate reports into the medical record. In technology’s most simple form, patients can be called or emailed symptom questionnaires to complete. This can be done while patients are in the hospital or waiting room, yet an important application is for longitudinal symptom tracking while patients are at home. Systems must be reliable, accurate, and permit efficient collection of symptom and health-related quality of life (HRQOL) data.

Electronic systems, usually employing a handheld computer and touch screen, are quite acceptable to patients [26,27,28,29]. While relatively little comparison of technology-based versus paper-based modes of administration has been conducted, one study found data equivalence between app-based and other delivery methods [30]. Another (*n* = 19) study of children with or without a speech or voice disorder also found that the scores did not differ between paper and pencil and electronic handheld device groups. However, the percentage of children who made answering errors or omissions was significantly smaller in the group who used an electronic handheld device. The device also permitted more efficient collection and manipulation of data [31].

Increasing in complexity, survey results can be fed back to the research team and physician in real time. Electronic methods of collecting data from patients have also allowed the presentation of real-time symptom and HRQOL measurements to clinicians, at the time of the clinical encounter with the patient. Feedback to clinicians is viewed favorably by patients [32,33] and clinicians alike [33]. Moreover, this strategy is feasible in busy oncology practices [34], does not increase consultation time, facilitates patient-clinician communication about symptoms or HRQOL, and increases the likelihood that symptom issues are addressed [33,35,36,37,38]. Many technology-based PRO collection systems permit assessment of symptoms and HRQOL in between clinic visits, at times that the clinician would not otherwise understand the patient’s condition. This is especially important since patients may be unwilling to call the office, and routine PRO assessment may provide opportunities to attend to symptoms before they escalate.

## 3. Assessment of Child Symptoms and Health-Related Quality of Life

### The Science of Self and Proxy Reporting

Accurate symptom reporting, timely assessment, and expert treatment are critical to preventing additional decrements to the patient’s physical, psychological, emotional, social, and existential domains of heath. Child self-report is considered the gold standard for assessing symptom burden and HRQOL, as parents and clinicians tend to under-report the severity and number of symptoms children are experiencing [39,40]. Discrepancies among reporters have been demonstrated in several disease groups, including children with cancer, congenital cardiac disease, and those who are status-post bone marrow transplantation [41,42,43]; however, the direction of this association can vary. For example, parents of children with sickle cell disease have been shown to give worse ratings of their child’s HRQOL compared with the child’s ratings [44].

In general, concordance is better (informant discrepancy is less) with physical symptoms compared with psychological symptoms [45,46]. This difference was recently demonstrated to be true in a population of children who received a pediatric palliative care consult and may be explained by the fact that physical symptoms are more readily assessed based on biomedical or behavioral changes [47]. Parent ratings can also be affected by their own life experiences, mental state, and distress levels [43,45,48,49,50]. Considerations around use of child self-report versus parent or clinician proxy-report are summarized in Table 1. In the end, the best approach to differing perspectives is to take advantage of their diversity, incorporating all viewpoints to provide a more global picture of the child’s experience [43].

Since child self-report is taken to be the gold standard when it comes to assessing their symptoms, novel methods are needed to obtain symptom assessments from children of all ages and developmental stages, validate additional pediatric measures across a wider variety of patient populations, understand patient-reported outcomes, translate results to the treating teams, and change supportive care therapies when warranted.

## 4. Patient-Reported Outcomes Assessment

A patient-reported outcome (PRO) is “any report of the status of a patient’s health condition that comes directly from the patient, without interpretation of the patient’s response by a clinician or anyone else” [51]. Medical outcomes have historically focused on biomedical or physiologic endpoints (e.g., progression free survival in oncology, overall survival). However, these outcomes do not capture a person’s experience of living with a medical condition or illness, such as side effects of the illness and its treatment, and treatment burden. To gain a better understanding of a patient’s lived experience, assessment of PRO that capture outcomes such as functional status, as well as aspects of HRQOL, including physical symptoms and functioning, emotional symptoms and social functioning are imperative. Adult oncologists endorse the utility of PRO collection, with clinical measures such as performance status being most important. PRO becomes even more important when multiple treatments options are under consideration, in the setting of advanced or incurable disease, or for patients receiving palliative care [52].

In recent years, systematic collection of PRO has also become an integral part of the assessment of new therapies and interventions [51,53]. Basch and colleagues demonstrated that patient self-reports of symptomatic adverse events (toxicities) were highly feasible (overall adherence of 93%) in a large, multicenter trial of 361 adults with cancer. Moreover, participants reported more toxicities than did investigators, indicating that this collection strategy utilizing PRO may enhance the precision of symptomatic toxicity collection in trials [54]. In both the clinical and research settings, PRO assessment is advantageous to patients, practitioners, and investigators alike, and therefore, integration of PRO assessment into clinical workflows and investigations will likely accelerate.

### Patient-Reported Outcomes Assessment with Feedback

With regard to clinical outcomes, feedback of PRO to clinicians in the aforementioned study conducted by Basch and colleagues resulted in fewer emergency room visits and hospitalizations [55]. Feedback also resulted in improved survival [56]. Whether this was due to improved symptom control and mitigation/prevention of worsening toxicity or enhanced tolerance of continued treatment warrants additional study. PRO data can improve care delivered by informing patient and clinician expectations, catalyzing communication about important issues, and promoting shared decision-making such that interventions meeting a patient’s specific needs and goals are chosen. Recent experience with PRO reporting to clinicians in a large health care system also suggests that it enhances physician satisfaction and prevents burnout by enhancing physician-patient relationships, increasing workflow efficiency and enabling crucial conversations that otherwise may not occur [57]. Suggestions for PRO assessment and feedback to clinicians are presented in Table 2.

## 5. Patient-Reported Outcomes in Pediatrics

Collection of PRO in pediatrics lags-behind the adult realm in both the clinical and research realm. For example, within the research realm, a review of clinical trials listed in ClinicalTrials.gov [61] and involving children with chronic conditions revealed that only 36 of 495 trials included patient and family-important outcomes, with these outcomes being particularly rare in drug trial and early phase trials [62]. The National Cancer Institute’s Common Terminology Criteria for Adverse Events (CTCAE) is a well-established system by which adverse events are gleaned from the medical record, potentially missing subjective symptoms that are reported by the patient and not reliably contained in the medical record. The Pediatric Patient-Reported Outcomes version of the CTCAE, or PRO-CTCAE, builds on the established CTCAE, allowing the child’s (or proxy’s) voice to be a routine trial endpoint. While not yet publicly available, the hope is that through the Pediatric- and Proxy PRO-CTCAE measures, the impact of an investigational treatment on a child can be more fully understood and addressed [63,64]. This continues to be a research- and clinical trial–focused tool rather than for use in routine clinical care settings.

Regarding clinical use, pediatric oncologists report that routine assessment of PRO would be useful in their practice, particularly those focused on pain, feeling sad or depressed, overall physical symptoms, problems with therapy adherence, and emotional issues [65]. When PROs are collected frequently and routinely, the process is normalized. It conveys a message to patients and families that their perspectives and experiences are valued. This, of course, means that clinicians must respond to PRO collected data in the clinical setting so that child reports are not ignored.

### Pediatric Patient-Reported Outcome Instrumentation

Pediatric PRO collection has historically been challenged by the number of instruments developed for collection of PRO from children as well as their inconsistent use. Many of these instruments are not based on a similar conceptual foundation (i.e., instrument content and domains are founded on different conceptual frameworks). Pediatric PRO instruments have also been developed with the use of different psychometric methods and properties (e.g., cross-sectional versus longitudinal). As a result, a wide range of instruments exist, with varying degrees of reliability, validity, and comparability [66]. Recent systematic reviews assessing PROs for children with cancer confirmed that substantial heterogeneity exists in content and distribution of items (e.g., which aspects of health are assessed and whether individual questions focus on function versus well-being) [67,68].

To address these issues, the National Institutes of Health (NIH)-sponsored PROMIS (Patient-Reported Outcomes Measurement Information System) measures, including pediatrics-specific measures, were developed in recent years as part of the NIH Roadmap for Medical Research Initiative [69,70,71,72]. Publicly available, PROMIS measures assess domains such as physical, emotional, and social health as well as symptoms (e.g., pain). PROMIS measures are designed to assess symptoms and function in the general population and in individuals living with chronic conditions with a brief completion time. Pediatric self-report PROMIS measures are available for ages 8–17 years and parent-proxy versions are available for children aged 5–17 years, in English, Spanish, and other languages. They are psychometrically sound, having undergone extensive testing using modern test theory, in both cross-sectional and longitudinal analyses (e.g., responsiveness, test-retest reliability) [73,74]. In addition, short forms and compatibility with computer adaptive testing make them highly usable. An additional strength of the PROMIS measures is that child and adult emotional distress measures are linked, allowing both populations to be evaluated within studies or for children to be followed into adulthood in longitudinal studies [75].

An innovative technological approach to instrumentation and collection of PRO is that of computer adaptive technology (CAT). In CAT, each question presented is selected based on a respondent’s previous response. This permits generation of an accurate score based on fewer numbers of questions than standard item administration would require. Developed in Europe, the Kids-CAT system has been validated as a system for measuring physical and psychological well-being, parent relations, social support and peers, and school well-being in children with asthma, diabetes, or rheumatoid arthritis. In addition to providing greater measurement precision and lower test burden compared to conventional tests, it provides immediate feedback reports to clinicians [76,77,78].

## 6. Novel Tools, Apps, and Toys for Symptom Assessment and Management

### 6.1. Online or Computer-Based Tools

Online tools are being developed both in a research context, and also in a patient-focused manner. One example of a research-focused tool is PediQUEST [5]. Evaluated in the first randomized controlled trial of a supportive care intervention for children with advanced cancer, PediQUEST is a web-based PRO data collection system that prospectively and longitudinally collected child self-reported and parent proxy-reported symptoms and HRQOL at three large cancer centers [79]. The PediQUEST study demonstrated that collection of PRO from children with advanced cancer was highly feasible. For example, among 708 potential child administrations (i.e., assessments of children old enough to self-report), 98% were provided a report [80]. This is of particular importance given that child participants had advanced illness. When reports were sent to clinicians, both parents and clinicians valued the reports. Reports facilitated communication about symptoms and HRQOL and resulted in improved emotional symptoms and well-being [5].

KLIK (Kwaliteit van Leven In Kaart (Quality of Life in Clinical Practice) is another online tool for pediatric PRO reporting that was developed in the Netherlands, and evaluated in large studies with thousands of patients [81,82]. The KLIK website generates an e-mail several days before an appointment that allows children (8–18 years old) or parents (for children age 0–7 years old) to complete PRO measures [81]. KLIK transforms child responses into an ePROfile which is shared with the child’s health care provider via a central website [83]. In a randomized multicenter study of children with juvenile idiopathic arthritis, reports were reviewed during a routine appointment (intervention group). In this group, psychosocial topics were discussed more often, and clinicians were more satisfied with the care provided [82]. Similar studies in the pediatric oncology population also revealed that psychosocial issues were more frequently raised among those who received the reports, without increasing the duration of the clinical encounter [84,85]. Of note, participants were prompted by email to complete assessments, suggesting that, at least in these pediatric populations, remote (i.e., not clinic-based) assessments are feasible.

Other online tools have been marketed in a direct to consumer fashion. My Quality (My Quality, Ltd, United Kingdom) is an online tool that allows families with children with life-limiting illness to designate and monitor parameters that they identify as impacting their quality of life [86]. Initial testing of the site revealed that it was highly usable and feasible, and the site’s graphic representation of change over time facilitated collaboration in the child’s care. Moreover, families who used the site had greater empowerment over time [87].

### 6.2. Mobile Apps

An increasing number of mobile apps designed for smart phones have also been developed for symptom tracking, symptom reporting, and symptom management, with some combining multiple focuses. Apps can be used by parents to report their young or non-verbal child’s symptoms. Apps are particularly attractive for adolescent and young adults, who may not reliably report their symptoms in clinic, notably around sensitive topics such as sexual dysfunction or body image. Some apps are focused on one symptom, such as PainSquad+ for pain [16] or SyMon-SAYS for fatigue [8], while others such as Symple (Symple Health, Andover, United Kingdom) may assess a broader range of symptoms, including fatigue, nausea, and mental health issues [88]. Another smartphone app allows tracking of child pain and interference caused by pain while simultaneously tracking parent response to their pain. Such a strategy allows analysis of the inter-relationships between the child’s pain experience and caregiver responses, such as how a child’s pain and pain interference changes after a given type of caregiver responses (protective versus minimizing responses) and whether caregiver responses to pain is predicted by child factors, pain, or caregiver mood [89]. The validated measures utilized within these apps are varied, making it difficult to compare one to another. However, instituting app-based symptom tracking within a clinic or small group of providers is an area ripe for quality improvement research.

More advanced apps, such as Pain Buddy [17], are starting to couple assessment with analysis and targeted intervention. Instead of solely tracking symptoms and distress over time, apps now have the capability to generate reports, send alerts to physicians, and deliver a focused intervention to the user. This allows a physician to plan for pharmacologic therapies and the user to receive immediate non-pharmacologic treatment, including mindfulness or breathing techniques. Other publicly available apps designed for children and adolescents manage symptoms using non-pharmacologic therapies and by teaching coping skills for managing pain, symptoms ,and stress [90]. Some examples are Healing Buddies Comfort Kit (Children’s Hospitals and Clinics of Minnesota, Ridgeview Medical Center, and DesignWise Medical, Version 1.1, Minneapolis, MN, USA) [91], Mindfulness for Children (Mindfulness for Children, Version 1.2, Niva, Denmark) [92], and Positive Penguins [93] (HR INSIDE PTY LTD, Version 2.0, Melbourne, Australia).

### 6.3. Therapeutic Toys

One emerging area in pain management for children is the use of therapeutic toys. Toys are a fun incentive and can promote social, emotional, and intellectual development [94]. In medicine, toys and play have often been employed as a method of distraction during procedures, such as suturing or IV sticks, or to promote education, such as dolls to prepare children for port-a-cath or gastrostomy tube placement.

As toy technology advances, they can also be utilized as a therapeutic alternative, helping to reduce pain, stress, fear, and anxiety. Some hospitals are already utilizing advanced sensory rooms or machines which engage a patient’s senses—smell, touch, sight, and sound. This can assist in having a calming effect and provide symptom relief without utilizing medication. Mixed-media toys may have both a physical toy and a mobile app that pairs with the toy to vibrate, feel emotions, and transport children to a soothing soundscape. One example is Jerry the Bear, a comforting companion for children with type 1 diabetes (Sproutel, Inc, Version 1.2.0, Providence, RI, USA) [95]. Similar comforting companions and apps can be designed to entertain, comfort, educate, connect, and mimic a child’s experience.

While there are many limitations and practical challenges in using web-based mobile apps and toys to assess symptoms and improve a child’s quality of life, this is also an opportunity to involve patients and families in developing technology that is user-friendly. Children are incredibly creative and technology-savvy. Engaging children around the development of these programs, apps, and toys may empower them to take control over their symptoms by providing valuable perspectives on living through illness, hospitalizations, and procedures.

## 7. Meeting Patients Where They Are: Integration of Palliative Care in the Clinic Setting to Address Symptoms

Integration of palliative care principles and services into routine outpatient care for children with high-risk cancer disease is associated with improved symptom management for patients and quality of life (QOL) for both children and families [19,96,97,98,99,100,101,102,103]. Adult cancer centers have already demonstrated that integrating palliative care into the care of patients with cancer improved patient’s pain, fatigue, depression, anxiety, and sleep [104,105]. Currently, this model is becoming recognized in pediatrics [19,99,106]. Several oncology or palliative care clinics have integrated web-based, computerized symptom assessments and PROs with validated tools [107]. For example, the Memorial Symptoms Assessment Scale, Pediatric Quality of Life inventory, and PROMIS forms have been utilized in both a clinical context and in research studies [5,17].

Perhaps most importantly, an interdisciplinary team-based assessment of a patient and family often yields additional information that cannot be gleaned from a survey. While integration or embedding within specialty clinics, such as oncology or cardiology, can be difficult and logistically challenging, there is value to a specialty palliative care team assessing, treating, and following the patient and family over time [19,98]. While a physician, nurse practitioner or nurse is extremely valuable in symptom assessment and treatment, the perspective of other team members should not be discounted. In fact, it is the experience of many palliative care teams that the social worker, chaplain, psychologist, child life specialist, or clinical pharmacist can be incredibly beneficial in assessing and managing physical symptoms, psychological symptoms, spiritual distress, and psychosocial pain [108,109,110,111]. The rationale for this is that many discussions are not about the symptom itself, but the meaning behind the symptom. This can include uncertainty about the cause or the future and whether this means diminished function [112]. When a patient’s concern about a symptom is routed to a medical provider, a physician tends to give scientific medical responses, while the patient may desire a listening ear, reassurance and empathy. See Table 3 for an overview of the symptom assessment methods.

## 8. Discussion

Recent innovations in addressing symptoms hold great promise in improving the care of children with serious illness. PROs, computer-based mobile apps, and toys are innovative solutions to the challenge of accurate pediatric symptom assessment and management. At the same time, they highlight areas in which future efforts are needed to advance care. One such area is that of symptom and HRQOL assessment methodology. Appreciation for the way in which a global view of a child’s experience is best obtained by self-report in conjunction with proxy-report is mounting. However, this must be accompanied by better understanding of what drives differences between these reporters, and how each perspective can best be understood in the context of the child’s total experience. As described in this paper, further instrument development is also imperative to improve psychometrics and standardization. With regard to the latter, this will require some consensus pertaining to the most important outcomes to capture. Within palliative care, PRO research that captures a child’s end-of-life experience is a particular need. Challenges to such work do exist; the child may be too sick to self-report during times of very advanced illness and highly emotionally charged circumstances. That being said, sensitivity, combined with a rigorous approach to this research, can overcome these challenges.

While still relatively early in development, systems for eliciting symptom and HRQOL data exist to facilitate efficient collection of longitudinal data and optimal medical care. Through technology-based collection of PRO, care is enhanced, including shared decision-making, effective communication and patient-centered care. At the same time we must guard against the possibility of moving from “high touch” to “high tech” care. Electronic medical records are gradually becoming more compatible with routine PRO assessment, allowing integration of reports. If PRO assessments are not tied to clinical encounters, systems and resources to address patient reports outside of clinical appointments in a timely manner will also be necessary.

We are just now scratching the surface of other PRO applications that extend beyond the point of care, such as evaluation of care quality, effectiveness, and value. These aspects of care have historically been measured in terms of care processes or downstream outcomes (e.g., readmission rates). In this age of value-based medicine, reimbursement is increasingly tied to patient outcomes as opposed to procedure or visit-based reimbursement. With value-based care placing greater emphasis on patient experience, such as patient well-being, now is the time for the incorporation of standardized measurement of PRO. PRO can thus become important indicators (performance measures) of provider and organizational performance, complementing other performance measures based in clinical outcomes and health care processes. Use of PRO in this regard further highlights the need to integrate PRO collection into electronic medical records.

Availing ourselves of the potential opportunities afforded by PRO assessment will necessitate overcoming barriers. First, optimal practices with regard to electronic administration of assessments (e.g., online versus app-based) must be delineated. The chosen methods must possess protections for patient privacy and confidentiality. Ideally these methods will also remain viable and compatible in the face of rapidly changing technology. Clinician buy-in to use these technologies can be achieved through involvement of clinicians in the development of PRO assessment practices as well as clinician education and staff training for maximum efficiency. A greater challenge may be organizational in nature. For example, a recent survey of pediatricians from 52 countries revealed largely organizational barriers to their assessment and use in clinical practice, including time, insufficient staff, logistical challenges, and financial resources [65].

Similar barriers will exist to more widespread use of computer-based or mobile apps. As patients, families, and clinicians have an increasing variety and number of choices to track and manage symptoms, it will become difficult to understand the measures, science, and methods behind each app. It would also become impractical for a physician to link with patients across multiple different sites. In starting to use a technology, collaboration with industry partners and information technology services may be needed to assist with installation and patient/clinician efficiency. Once mobile utilization has started, continual effort will be required to limit practice variation through quality improvement research and initiatives.

While all of the former methods are highly reliant upon technology incorporation into standard clinical practice, routinely integrating pediatric palliative care into the care of children will shift the culture of care to one emphasizing comfort and quality of life, regardless of treatment plan or outcome. Despite an increasing body of literature supporting pediatric palliative care integration as a standard of care for many children with complex, chronic, and life-threatening illness, utilization in practice has been slow. Several limitations remain to better integration including the size of the pediatric palliative care workforce, the comfort and knowledge of the pediatric clinicians, the system-level support, a billing infrastructure that values quality, and the ability to collaborate with local and state community organizations. When palliative care is better integrated, the lives of patients and families can be improved by focusing on symptom control, enhancing quality of life, and communicating well to enrich care coordination. Pediatric palliative care consultation combined with patient PRO may be an even more powerful approach to improving child HRQOL and should be investigated.

To the degree that disease-based screening, assessments, and treatments have improved over the years, the same attention needs to be paid to the patient experience. Novel approaches to improve the reporting of symptoms by patients and families, and the interpretation and management skills of clinicians are on the horizon. Yet, continued development and study of technological and team-based methods to improve symptom assessment and control are needed to drive palliative care forward.

## Figures and Tables

**Table 1:** Considerations around use of self, parent and clinician report when assessing child symptoms and health-related quality-of-life (HRQOL).

Self-Report	Parent Report	Clinician Report
For subjective outcomes (e.g., symptoms, HRQOL), the person experiencing the outcomes is the expert. Thus, self-report is considered the gold standard	Parent often has a longstanding and nuanced knowledge of child; besides the child, is often considered “the expert” regarding the child’s experience	May or may not be familiar with child, which can impact ratings
Various factors (e.g., medical, developmental, cognitive) may influence self-report	When child unable to self-report, parent report is often considered the next best alternative	Valuable when the child cannot self-report due to developmental considerations or illness
Young and seriously ill children may be limited in their ability to self-report at all	In general, greater agreement with child ratings for observable functioning (e.g., physical symptoms and HRQOL) and less for functioning that cannot be observed (e.g., emotional symptoms and HRQOL)	Like all raters, reports may be colored by the rater’s own experiences, beliefs, skill level, academic interests, expectations and points of reference
	May be influenced by parent factors, such as parent anxiety or distress (generally associated with worse ratings) as well as parent expectations and points of reference	Clinicians may have the experience of caring for many children under similar circumstances, which can shape their views about a particular child’s experience
	Parent proxies can also provide input regarding the parent and family experience	

**Table 2:** Suggestions for use of patient-reported outcomes (PRO) and feedback reports in the clinical setting.

Aspects of Patient-Reported Outcomes and Feedback Reports	Considerations
Choice of measures and outcomes	Outcomes should be meaningful and important to the patient.
	When possible, select standardized measures.
	Utilize instruments that have been evaluated by members of the target audience.
	If available and pending context (research vs. clinical), consider use of condition-specific measures which may be more sensitive to intervention effects.
	Carefully select frequency and timing of assessments to avoid survey fatigue, ensure that assessment points are clinically meaningful and provide results that can be acted upon in future clinical visits.
Data presentation	Make displays intuitive using pie graphs and line charts showing trends in function and symptoms. Make reports easily accessible to viewers [58].
	Present a defined, carefully selected set of data (avoid presenting too much).
	Present current scores and recent trends. Correlation of trends with recent clinical events (e.g., chemotherapy, hospitalization) is also helpful.
	Make scores easy to interpret for patients and clinicians. Provide context for clinicians who may not be familiar with symptom or HRQOL scores and meaningful changes in scores. Other strategies to effectively present results to patients and clinicians have recently been described [59,60].
Clinician Use	Make reports available at the point of care in electronic format via website, emailed to clinicians, and incorporated within the electronic medical record. The optimal mode will depend upon the clinical practice.
	Link reports to supportive care guidelines/recommendations for intervention
	Minimize disruption to the clinical workflow.
Implementation	Ensure buy-in from patients/families and clinicians alike when embarking on the study.
	Ensure patient interface is easy to use.
	Minimize time burden for all users.
	Ensure processes for responding to reports in a timely manner.
	Ensure adequate networks, software and properly configured devices for data collection.

**Table 3:** Methods of pediatric symptom assessment.

Assessment	Strengths	Weaknesses	Application to PPC
Patient-reported outcomes (PROs)	Can be adapted to a number of electronic interfaces, including EMR and direct-to-clinician reports Variety of choices for different symptoms, HRQOL measures Valid and reliable measures for pediatric research studies Can be fed back to clinical team for improved symptom management	Long forms can be time-consuming Need to consider child self-report vs proxy report PROs lacking for non-malignant conditions and within pediatric research Limitation of PROs in patients at end-of life, or who are non-verbal	Can be incorporated prior to and within symptom management visits PROs can be completed at home, with hospice providers and sent to hospital-based team Allows for multi-site research studies
Online tools (e.g., KLIK, PediQUEST, MyQuality) [5,80,81,83,86]	Utilize a variety of PROs Applicable in research and clinical settings Surveys prior to physician appointments increased psychosocial discussions Improves parent and clinician satisfaction	Initial investment into development and technology Need for interface between electronic assessments and EMR	Allows families to choose the measures of importance Easier communication with busy clinicians Feasible for children with advanced disease
Mobile apps	Can be used by parent, child or both allowing inter-relationship analysis across a variety of symptoms Direct reporting to clinicians Ability to provide targeted interventions focusing on non-pharmacologic therapies	Measures within apps vary, making it hard for clinicians to understand results or compare across apps Availability only on some operating systems (e.g., Apple (Apple Inc., Cupertino, CA, USA) vs Android (Google, Mountain View, CA, USA))	Teaches and enhances patient coping skills Ability to teach mindfulness, guided imagery, and breathing techniques Applicable for research on pain, fatigue, etc.
Therapeutic toys	Promote social, emotional development Can reduce pain, stress, fear Mixed-media capability with toy and mobile app Engaged children who can assist in design ideas	Can be expensive to acquire for hospitals or patients/parents Toys must meet many hospital safety and compliance regulations Few options available	Utilized as distraction for procedures Engages a patient’s senses of smell, touch Children can use toys to communicate emotions and feelings
Interdisciplinary Pediatric Palliative Care team	Ease of assessment in inpatient and outpatient settings Benefit of multiple member assessment (physician, nursing, social work, chaplain, child life) Data supporting improved patient/family outcomes	Less feasible when patients are home Increased personnel and time needed Not available at all pediatric centers	Provides human connection for families Have ability to combine with any other strategy Provides medical opinion and puts treatment plan in place

EMR: electronic medical record, HRQOL: health-related quality of life.
